# Sustained release of the candidate antiretroviral peptides T-1249 and JNJ54310516-AFP from a rod insert vaginal ring

**DOI:** 10.1007/s13346-015-0273-8

**Published:** 2016-01-19

**Authors:** Diarmaid J. Murphy, Katie Amssoms, Geert Pille, Aileen Clarke, Marc O’Hara, Jens van Roey, R. Karl Malcolm

**Affiliations:** School of Pharmacy, Queen’s University Belfast, Belfast, BT9 7BL UK; Janssen Research and Development, Turnhoutseweg 30, 2340 Beerse, Belgium

**Keywords:** HIV-1 microbicide, Silicone elastomer vaginal ring, Rod insert ring

## Abstract

Administration of biomacromolecular drugs in effective quantities from conventional vaginal rings is hampered by poor drug permeability in the polymers from which rings are commonly constructed. Here, we report the formulation development and testing of rod insert rings for sustained release of the candidate antiretroviral peptides T-1249 and JNJ54310516-AFP (JNJ peptide), both of which have potential as HIV microbicides. Rod inserts were prepared comprising antiviral peptides T-1249 or JNJ peptide in combination with a hydrophilic excipient (sodium chloride, sodium glutamate, lactose or zinc acetate) dispersed at different loadings within a medical grade silicone elastomer. The inserts were tested for weight change and swelling when immersed in simulated vaginal fluid (SVF). Dye migration into the inserts was also assessed visually over 28 days. In vitro release of T-1249 and JNJ peptide from rings containing various insert types was tested. Weight change and degree of swelling of rods immersed in SVF was dependent on the type and concentration of excipient present. The rods displayed the following rank order in terms of weight change: sodium glutamate > zinc acetate ≈ sodium chloride > lactose. The weight change and degree of swelling of the inserts did not correlate with the level of dye uptake observed. In vitro release of T-1249 was improved through addition of lactose, sodium chloride and sodium glutamate, while release of JNJ peptide was improved through addition of sodium chloride or sodium glutamate. Sustained release of hydrophobic peptides can be achieved using a rod insert ring design formulated to include a hydrophilic excipient. Release rates were dependent upon the type of excipient used. The degree of release improvement with different inserts partially reflects their ability to imbibe surrounding fluid and swell in aqueous environments.

## Introduction

Vaginal rings delivering small molecule antiretrovirals are currently attracting interest as a long-acting strategy for reducing heterosexual transmission of human immunodeficiency virus type 1 (HIV-1) [1–4]. For example, a dapivirine-releasing ring is currently undergoing phase III clinical testing in Africa [5, 6]. The potential benefits of vaginal rings over conventional vaginal formulations e.g. gels and creams, include coital independence, improved adherence and the ability to deliver microbicide compound(s) over extended time periods [1, 3, 7]. Results from the CAPRISA trial highlighted the impact that user non-adherence can have on microbicide effectiveness [8]. For a successful microbicide strategy to be effective in widespread use, it should be easy to use and be able to deliver microbicides at sufficient rates to ensure protection over a minimum period of time [7].

Marketed vaginal rings are typically composed of hydrophobic polymers, such as silicone elastomer or ethylene vinyl acetate copolymer, with the crystalline drug substances either homogeneously dispersed throughout the ring (a matrix-type design) or located in a drug-loaded central core which is covered by a rate-controlling polymer membrane (a reservoir-type design). In both designs, drug release is dependent upon permeation of the drug through the polymer network, and therefore requires a degree of drug solubility and diffusivity within the polymer [7, 9, 10]. This drug permeation mechanism generally lends itself to daily release rates within the low microgram to low milligram range for small hydrophobic molecules when formulated within hydrophobic polymers. By contrast, large and/or hydrophilic molecules tend to exhibit very poor release characteristics. A number of high molecular weight compounds are currently in development as potential microbicides, including anti-HIV antibodies, the CCR5 inhibitor chemokine analogue 5P12-RANTES and various peptide-based fusion inhibitors [11–13]. New ring formulations are therefore needed for effective vaginal administration of such molecules.

Attempts to improve the release of high molecular weight drugs from hydrophobic polymers by incorporation of hydrophilic excipients have been reported previously [14–16]. However, this approach is generally not applicable to vaginal rings owing to the very considerable swelling that occurs when large amounts of fluid are absorbed into the ring at the high excipient loadings required to give clinically significant release rates. Alternatively, loading very high concentrations of the high molecular weight drug into the ring device might offer enhanced release rates. However, the relative expense of most biomolecular drugs is a major constraint, and substantial burst effects would be anticipated.

**Fig. 1:**
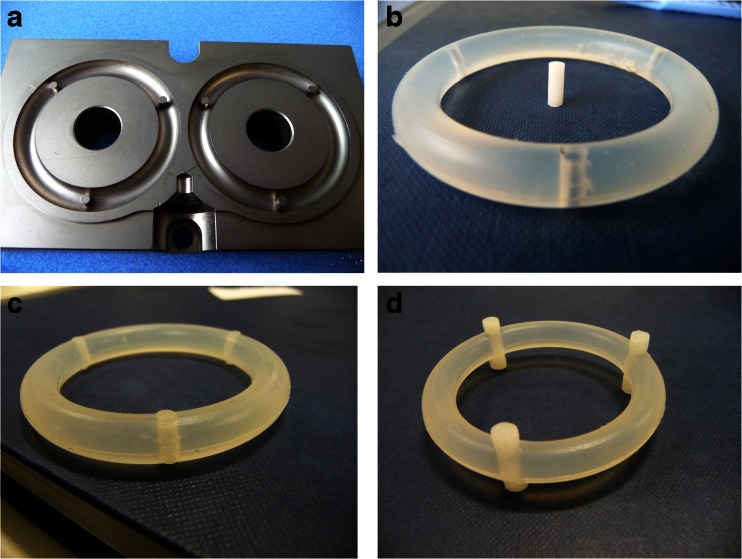
Photographs of the moulds used for rod insert ring production **(a)**, a rod insert and empty ring (**b**), the ring with inserts present **(c)** and a rod insert ring after 28 days on release in IPA:H_2_O **(d)**

Recently, we reported a rod insert vaginal ring design for sustained release of macromolecules, comprising a non-medicated silicone elastomer body with cavities for insertion of various rod-shaped drug carriers (Fig. 1) [17]. The benefits of this system include a greatly reduced amount of drug required per ring (as only the inserts contain the drug), and the ability to easily modify drug release through the use of hydrophilic excipients that swell and dissolve in contact with water. In addition, the high temperatures normally required in manufacture of the ring body can be avoided when formulating heat-sensitive drugs. Previous work has demonstrated that peptide- and protein-based drugs can be released from silicone-based rods with the release rate varied through hydrophilic excipient addition [18–24]. Other groups are also investigating similar modified ring strategies to deliver macromolecular actives [25, 26].

Here, we compare the impact of hydrophilic excipients on the release rates of two hydrophobic polypeptide microbicide candidates. T-1249 (molecular weight 5037 g/mol) is a 39-residue peptide that inhibits fusion of HIV to the host target cell by blocking the conformational change in the HIV envelope protein gp41 that is necessary for the fusion of the viral and the target cell membranes. JNJ54310516-AFP (JNJ peptide, molecular weight 4586 g/mol as TFA salt) is a 30-residue cholesterol-capped peptide.

## Materials and methods

### Materials

Platinum-catalysed, medical grade, silicone elastomer two-part kit (LSR9-9509-30) was supplied by NuSil Silicone Technology Inc. (Carpinteria, USA). JNJ54310516-AFP was provided by Janssen Research and Development (Beerse, Belgium). T-1249 was obtained from Trimeris (Durham, USA). HPLC-grade acetonitrile and isopropanol (IPA), along with trifluoroacetic acid (TFA), sodium chloride (NaCl), sodium glutamate (NaGlu), zinc acetate (Zn(CH_2_COOH)_2_), lactose and methylene blue dye were obtained from Sigma-Aldrich (Gillingham, UK). NaCl, NaGlu, Zn(CH_2_COOH)_2_ and lactose were milled with a mortar and pestle and passed through a 180 μm-sieve before use. Medical grade PVC tubing was obtained from NuncNalgene (NY, USA). Simulated vaginal fluid (pH 4.2; SVF) was prepared using analytical-grade reagents according to a previously described recipe [27]. HPLC-grade water was obtained using a Millipore Direct-Q 3 UV Ultrapure Water System (Watford, UK).

### Covered rods and rod insert manufacture

Dispersed mixtures (total weight 2.0 g) comprising Parts A and B LSR9-9508-30 addition cure silicone elastomer mix (1:1) and from 0 to 50 % *w/w* of one of NaCl, NaGlu, Zn(CH_3_COO)_2_ or lactose were prepared using a DAC 150 FVZ-K Speedmixer (30 s, 3000 rpm). The mixtures were injected into medical grade PVC tubing (3.0 or 4.0 mm internal diameter) via a disposable plastic syringe and allowed to cure for at least 24 h at room temperature before being cut into 7.6-mm length sections to produce covered rods. Peptide-loaded rod inserts were manufactured in a similar fashion incorporating either 20 or 40 % *w/w* excipient and 1 % *w/w* peptide. The PVC tubing was removed entirely upon curing for peptide-loaded rod inserts.

### Rod insert ring manufacture

Parts A and B LSR9-9508-30 addition cure silicone elastomer mix were mixed in equal quantities before injecting onto specifically designed moulds on a pneumatic, electrically heated, laboratory-scale, ring-making machine. The moulds used for rod insert rings incorporate three pins that create a 3.0-mm wide bore at points evenly spaced around the ring (Fig. 1). Heating at 80 °C for 3 min cured the rings. Ring dimensions were 56.5 and 7.6 mm overall and cross-sectional diameter, respectively; with insert dimensions 7.6 × 3.0-mm length and cross-sectional diameter, respectively.

### Swelling and dye uptake testing of drug-free PVC-covered rod inserts

Covered rods were immersed in 10 mL SVF. Weight change was assessed daily over the first 14 days and then on every other day through to day 28, with complete replacement of the media. Fluid ingression into the covered rods was investigated by immersing rods in 10 mL SVF containing 20 μg/mL methylene blue dye. Photographs of the rods were taken daily for 7 days and then weekly thereafter. Rod inserts were examined for appearance and defects using a Nikon TE300 ECLIPSE inverted microscope with image capture by a DXM1200 digital still camera using ACT-1 software for image processing.

### HPLC analysis

All analyses were conducted on a LUNA C18 5 μm column (150 × 4.6 mm). JNJ peptide was separated using a gradient method varying 0.05 % TFA in water (A) and 0.05 % TFA in acetonitrile (B) over 40 min from 95 % A, 5 % B to 10 % A, 90 % B. A flow rate of 1 mL/min and column temperature of 50 °C was maintained throughout with detection at 215 nm. Under these conditions, JNJ peptide exhibited a mean retention time of 25.6 min.

T-1249 was separated using a gradient method with detection at 220 nm. The total flow rate was maintained at 1 mL/min while the mobile phase components 0.1 % TFA in water (A) and 0.08 % TFA in ACN (B) were varied over 12 min from 57 % A, 43 % B to 53 % A, 47 % B. The column temperature was maintained at 30 °C throughout. T-1249 displayed a mean retention time of 5.4 min. A series of calibration standards was run with each set of release samples and the concentrations of peptide calculated from the standard curve run on the day. Linear regression of the combined standard curve data allowed calculation of the limit of detection and quantification according to the ICH Q2(R1) guidelines [28] displayed in Table 1.

**Table 1:** Limits of detection, quantification and correlation coefficient for T-1249 and JNJ peptide

Peptide	Release medium	Limit of detection (μg/mL)	Limit of quantification (μg/mL)	*r* ^2^
T-1249	IPA:H_2_O	0.45	1.36	0.9823
JNJ peptide	IPA:H_2_O	0.47	1.43	0.9804
JNJ peptide	SVF	0.60	1.81	0.9693

### In vitro release testing of rod insert vaginal rings

Rings were placed into 250-mL glass flasks with 30 mL of a 1:1 mixture of IPA and water (IPA:H_2_O). The first 2 days release of JNJ peptide into IPA:H_2_O were performed using 20 mL while the minimum volume required to allow complete insert swelling was established. Samples of the release media were taken daily for 14 days, and approximately every 2 days thereafter, with complete media replacement each time. Samples that could not be run on the day of analysis were stored at 4 °C. SVF is the release medium of choice for sufficiently soluble drugs as it mimics the normal vaginal fluid most closely. Given the relatively small loading of peptide in the rings and their poor aqueous solubility (<10 μg/mL in SVF), release into IPA:H_2_O was also performed for both peptides to allow easier detection and analysis, and for comparative purposes.

## Results

### Covered rod swelling and dye uptake

**Fig. 2:**
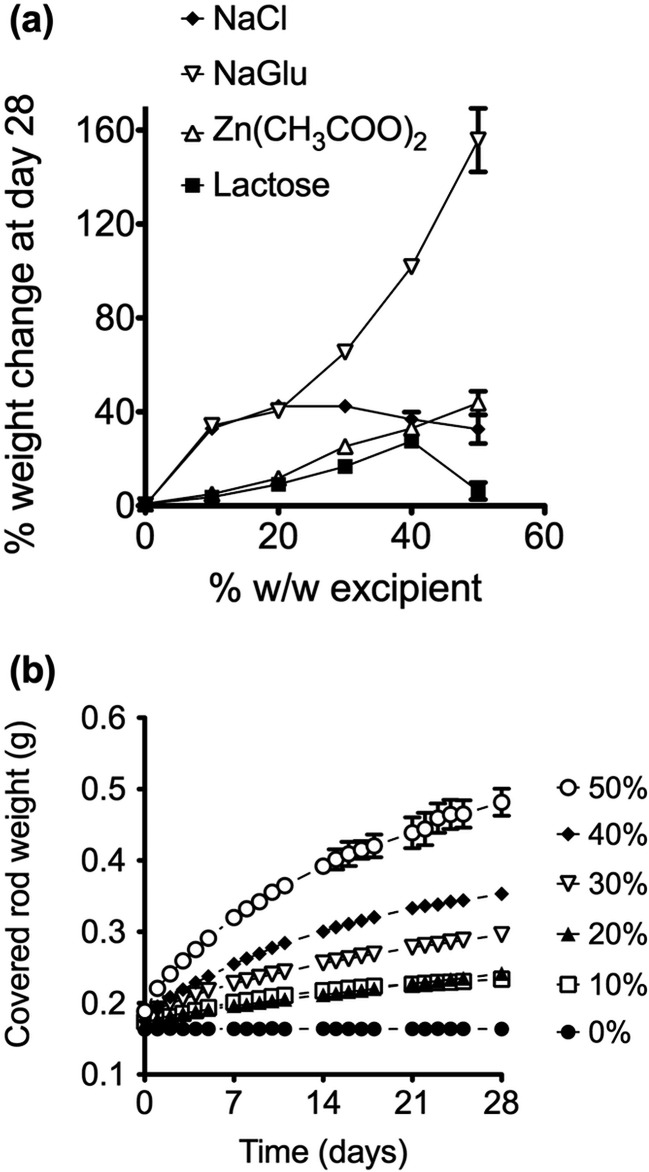
**a** Mean weight change of covered rods after 28 days by excipient type and loading. **b** Representative weight change occurring over 28 days for covered rods containing different amounts of NaGlu. All data points are the mean of four replicates with *error bars* denoting standard deviations

The mean weight change measured after different covered rods were immersed in SVF for 28 days is presented in Fig. 2a. NaGlu and Zn(CH_3_COO)_2_ both exhibited weight changes proportional to the amount of excipient incorporated into the covered rod. In contrast, NaCl displayed a maximal increase in the percentage weight change with 20 and 30 % *w/w* loadings. Higher loadings showed reduced percentage weight changes. Lactose displayed a proportional increase in weight change to excipient loading up to a loading of 40 % *w/w* with 50 % *w/w* rods displaying a very small percentage weight change by comparison, 27.5 vs. 6.3 %, respectively. NaGlu exhibited the largest percentage weight change while lactose exhibited the smallest. As a representative example, Fig. 2b shows the mean weight change of 3.0-mm covered rods over 28 days for all loadings of NaGlu. In contrast, covered rods with no excipient did not display any weight change over the 28-day period. As shown in Fig. 2b for NaGlu, weight changes occurred over 28 days for all of the covered rods.

Uptake of methylene blue dye in SVF (20 μg/mL) into covered rods was assessed periodically for each insert type to give a visual indication of the extent of swelling and fluid access. Representative photographs of covered rods containing each type of excipient at 40 % *w/w* loading are presented in Table 2. Differences in both the degree of swelling and the level of methylene blue dye staining were observed for the different inserts. For example, NaCl and NaGlu inserts displayed clearly observable swelling after 1 day of being immersed in SVF, while the lactose insert did not. Swelling was observed with all inserts by day 7. NaGlu exhibited the largest percentage weight gain over 28 days, five times greater than that observed with NaCl. However, NaCl-covered rods were more intensely coloured than equivalent NaGlu rods. A similar pattern was observed for the Zn(CH_3_COO)_2_ compared to lactose-containing covered rods. Again, the Zn(CH_3_COO)_2_ covered rods displayed a greater degree of weight gain but poorer methylene blue dye uptake. In terms of intensity of methylene blue dye staining, lactose and NaCl inserts showed the greatest level of colouration while Zn(CH_3_COO)_2_ inserts showed the lowest.

**Table 2:**
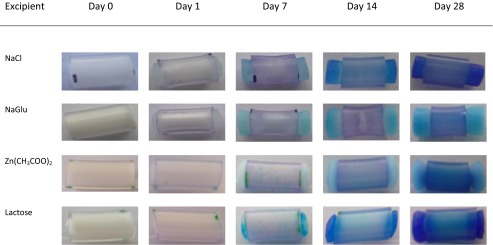
Ingress and swelling of covered rods made with 40 % *w/w* of each excipient over 28 days following immersion in SVF containing 20 μg/mL methylene blue dye

### In vitro release of T-1249 and JNJ peptide from rod insert rings

**Fig. 3:**
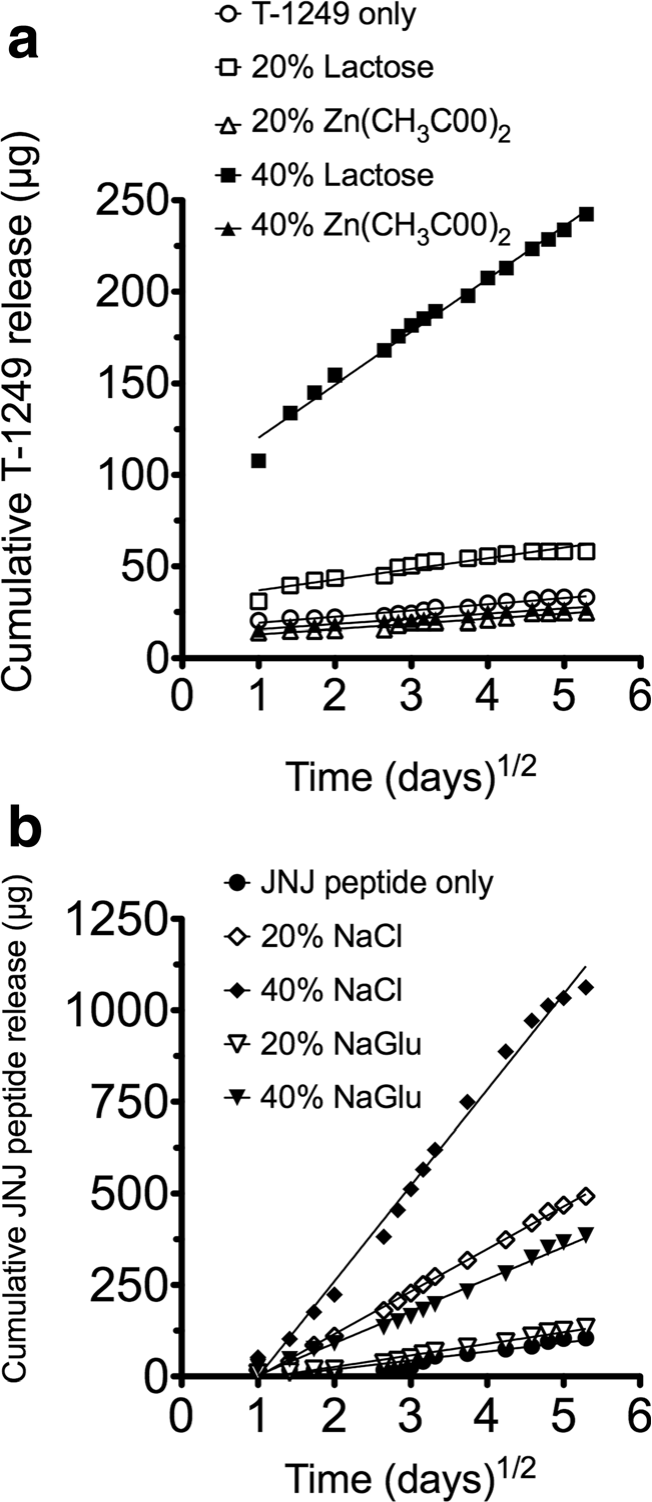
Cumulative release from rod insert rings of T-1249 with either 20 or 40 % lactose or Zn(CH_3_COO)_2_ **(a)** and JNJ peptide with 20 or 40 % NaCl or NaGlu **(b)**

Cumulative in vitro release data for T-1249 into IPA:H_2_O over 28 days from rod insert rings containing either lactose or Zn(CH_3_COO)_2_ at 20 and 40 % *w/w* are presented in Fig. 3a. Release of T-1249 was low for each of the formulations tested. Inserts containing 40 % *w/w* lactose showed a release rate that was approximately 10-fold faster than inserts with no excipient. Zn(CH_3_COO)_2_ did not improve release at either the 20 or 40 % *w/w* level. NaCl and NaGlu also improved release with 40 % NaCl providing the largest impact (data not shown).

The cumulative in vitro release of JNJ peptide into IPA:H_2_O measured over 28 days from rod insert rings containing either NaCl or NaGlu is displayed in Fig. 3b. Release of the peptide was improved by addition of either NaCl or NaGlu at both 20 and 40 % *w/w*.

Table 3 presents the gradient of the cumulative release lines with coefficient of determination values, the total amount of peptide released, the percentage released and the fold-increase in release calculated for each of the excipients. Without any excipient addition, release of both peptides from the inserts is minimal (2 and 6 % for T-1249 and JNJ peptide, respectively). However, for JNJ peptide, this increased to over 60 % released using 40 % *w/w* NaCl-loaded rods. A similar increase with NaCl addition was seen for T-1249 in terms of the gradient of release rate vs. root time, but the maximum percentage release was still less than 20 % of the theoretical loading.

**Table 3:** Release rates, coefficient of determination values, total peptide release and relative increase seen in the cumulative release of T-1249 and JNJ peptide into IPA:H_2_O plotted against the square root of time for different rod insert formulations

Formulation	Gradient of the cumulative release line^a^	*R* ^2^ value	Total peptide released (μg)^b^	% released^c^	Fold increase^a^
T-1249 only	3	0.9550	33	2	–
20 % Zn(CH_3_COO)_2_	3	0.9490	25	1	0
40 % Zn(CH_3_COO)_2_	3	0.9693	27	2	0
20 % lactose	6	0.9140	58	3	2
40 % lactose	29	0.9889	242	14	10
20 % NaCl	53	0.9804	236	14	18
40 % NaCl	44	0.9898	257	15	15
20 % NaGlu	10	0.9841	76	4	3
40 % NaGlu	15	0.9826	163	10	5
JNJ peptide only	24	0.8874	105	6	–
20 % NaCl	116	0.9978	493	29	5
40 % NaCl	261	0.9912	1063	62	11
20 % NaGlu	32	0.9770	133	8	1
40 % NaGlu	88	0.9898	385	23	4

^a^Values rounded to the nearest whole number

^b^Values quoted to the nearest μg

^c^The percentage peptide released was calculated based on a theoretical loading of 1705 μg per ring and quoted to the nearest per cent. This figure is 1 % of the mean weight of the total rod insert weight per ring, used in the peptide-only rings for the JNJ peptide. Weights for the T-1249 1 % loaded rod inserts are not available, so the mean weight of the total rod insert per ring for the peptide was used

## Discussion

To date, vaginal rings have mostly been used for delivery of small, highly potent hydrophobic actives. They are not normally considered for the release of macromolecules due to the extremely limited diffusion of large molecules through the polymer matrix. In addition, a large quantity of drug would be required to provide adequate loading for a human-sized ring. For highly potent macromolecular drugs, this may represent a very significant cost per ring with the majority of the material never released or recovered from the polymeric matrix. One possible strategy to overcome these problems is the rod insert ring [17]. Here, the ring design is modified to accommodate drug-loaded inserts that are made with excipients designed to promote release. We utilised the rod insert ring strategy in an attempt to improve the release of two peptides in development as microbicides, T-1249 and JNJ peptide, both of which are too large for delivery from a standard ring device. In the rod insert ring design, the choice of insert material and excipient is critical in determining the extent of release. We used rods composed of a similar elastomeric silicone used to manufacture rings and considered a number of excipients NaCl, NaGlu, lactose and Zn(CH_3_COOH)_2_. NaCl and NaGlu have previously been used to improve release from covered rods [14, 23]. Sugars such as sucrose and mannitol have also been considered in this context but lactose has not been investigated despite its widespread use in formulation as a diluent [18, 20] There is some evidence that zinc salts have antiviral effects, against both HIV and HSV [29–31], so we decided to investigate its potential to improve peptide release from the rod insert ring.

Osmotic pressure is thought to be a key driver for the release of components from silicone elastomers through pore and crack formation [20, 32, 33]. So, we initially considered the impact of different excipients on the swelling and fluid ingression behaviour of the insert. The weight of blank elastomeric rods immersed in SVF was measured daily over 28 days. The percentage weight change for each insert at day 28, displayed in Fig. 2a, demonstrates that incorporation of hydrophilic excipients can have a dramatic impact on the extent of fluid ingress into elastomeric rods that would otherwise remain largely unchanged in an aqueous environment. An example of the impact of excipient loading on swelling behaviour is displayed in Fig. 2b for NaGlu. This shows a large loading-dependent increase in the weight of the covered rod over 28 days. Surprisingly some covered rods showed a drop in swelling at higher concentrations of excipient e.g. compare lactose at 40 and 50 % *w/w* loading (Fig. 2a). It is not clear why there would be a reduction in swelling seen with an increase in salt or sugar inclusion. One possible explanation is that mixing causes aggregation of components or they get trapped within a silicone layer within the rod structure. This would effectively seal off certain parts of the rod from water ingression and may be a symptom of incomplete mixing.

The extent of methylene blue dye uptake into covered rods was also assessed visually by immersing the rods in a solution of the dye in SVF (Table 2). The level of methylene blue staining did not correlate with the degree of swelling observed in each case. For example, despite exhibiting the largest amount of swelling, NaGlu covered rods were amongst the most lightly coloured. Similarly, Zn(CH_3_COO)_2_ rods were also lightly coloured despite a reasonable degree of swelling. In contrast, NaCl-covered rods were intensely coloured as were lactose-covered rods despite minimal swelling. Methylene blue can form dimers and higher order aggregates with increasing dye concentration which impacts upon its spectral properties [34]. In addition, adsorption of dye to a surface can change its spectral properties [35]. Also, the extinction coefficients of methylene blue vary substantially depending on whether the monomer, dimer or trimer is considered. The covered rods offer the possibility of substantial dye adsorption as well as variation in the impact of the salt on dye spectral properties. Finally, it is possible that dye uptake was similar between inserts but appeared less intense due to the volume increase upon swelling. Combined, these factors may play a significant role in changing the relative intensity of the methylene blue staining observed with each rod insert. Further studies are required to separate and quantify these effects.

The effect of all excipients, at the 20 and 40 % *w/w* level, on the release of T-1249 from rod insert rings was assessed over 28 days (Table 3). Figure 3a presents data for the impact of Zn(CH_3_COO)_2_ and lactose on T-1249 release. This demonstrates how poor release is from a standard silicone elastomer system. It also shows that inserts containing Zn(CH_3_COO)_2_ are not effective at promoting release. In contrast, lactose-loaded rods exhibited a detectable increase in release rate at 20 %, and a large increase at 40 %. This is despite a similar level of insert swelling observed with both compounds up to the 40 % level. A substantial increase in release rate was also observed for rod inserts containing NaCl and NaGlu (Table 3). A similar impact on release rate was observed with 20 and 40 % NaCl-loaded rods, 18-fold vs. 15-fold, respectively, which is in line with the degree of swelling observed with blank rods, Fig. 2a.

In trying to relate the impact on release to the degree of swelling and or dye uptake, the data for T-1249 presents a mixed picture. Although NaCl does improve release approximately in line with the level of insert swelling observed, NaGlu showed a lower impact on release despite exhibiting greater swelling. Similarly, despite a similar impact on blank insert swelling, lactose exhibited a greater impact on release than Zn(CH_3_COO)_2_. These trends are more reflective of those seen with dye uptake in blank rods where a more intense dye staining was observed with NaCl and lactose inserts than equivalent inserts of NaGlu or Zn(CHCOO)_2_, Table 2. Zn(CH_3_COO)_2_ would be expected to produce a higher osmotic pressure than lactose, NaCl or NaGlu as it has three ion species per molecule dissolved compared to one for lactose and two for the other salts. However, T-1249 does contain more acidic than basic residues (8 vs. 4, respectively). At pH 4.2, these acidic residues would still be largely in the positively charged state, so it is possible that electrostatic effects associated with the use of Zn(CH_3_COO)_2_ serve to retard the release of T-1249. This does not explain the disparity between the release improvement observed with NaCl compared to NaGlu.

Approximately 3 μg T-1249 was released on day 28. This would correspond to a vaginal peptide concentration of approximately 40 nM, assuming similar in vivo release rates and maximal vaginal fluid and semen volumes of 8 mL each [27, 36]. With an IC_50_ value of approximately 20 nM against a wild-type HIV strain, this concentration is likely too low to offer protection against infection [37].

NaCl and NaGlu were tested for their impact on the release of JNJ peptide. NaGlu exhibited the largest degree of swelling with blank inserts and NaCl as well as displaying the best uptake of methylene blue also provided the largest improvement in release of T-1249. The cumulative in vitro release of JNJ peptide displayed in Fig. 3b shows that NaCl had a larger impact on peptide release than NaGlu at both concentrations tested, similar to the results seen with T-1249. In fact, 20 % NaCl-loaded rod inserts provided higher release rates of JNJ peptide compared to 40 % NaGlu loaded rods. From the values displayed in Table 3, it can be seen that 40 % NaCl inserts provided the greatest improvement in release (approximately 10-fold). Given a much better baseline release for this peptide compared to T-1249, this level of improvement is encouraging. Similar to the T-1249 experiment, the excipient that displayed the best swelling did not prove most effective at promoting peptide release. Rather, the excipient displaying better methylene blue uptake provided the greater increase in peptide release rates. The amount of peptide released per day at day 28 was approximately 15 μg. Again, a very rough estimate of vaginal fluid concentration can be calculated using the method outlined above for T-1249, yielding a predicted concentration of 204 nM. Data on file for this peptide give an EC_50_ value of 0.3 nM suggesting that this release rate may be sufficient to provide protection assuming the release seen in vivo matched that measured in vitro and with the same caveats regarding the vaginal fluid volumes mentioned above.

Comparing the release of T-1249 and JNJ peptide from the peptide-only inserts reveals a much greater release rate for JNJ peptide (33 vs. 105 μg peptide released, respectively). The cause of this difference in release rates is not clear. T-1249 is slightly larger than JNJ peptide and would hence be expected to display a slightly lower release rate, but the size of the difference in release suggests other factors are playing a role. Comparing the rates seen with the different excipients shows that NaCl is by some measure the most effective at promoting release. Lactose and sodium glutamate show a similar level of effectiveness, and Zn(CH_3_COO)_2_ has no impact. The very low release rates observed overall probably reflect the poor solubility of the peptides in aqueous media. However, this may not have the same relevance in vivo where there is constant fluid exchange and a large tissue sink for hydrophobic compounds.

The daily release values for both peptides after day 3 were generally at or below the limit of quantification for their assays (Table 1). However, the overall trends observed are relatively strong and demonstrate that large molecular weight peptides can be effectively released from a rod insert ring device. This allows very expensive and potentially thermally labile drugs to be incorporated into a modified vaginal ring at room temperature using smaller quantities than would be required to make a traditional matrix or reservoir-type device. The use of the insert also allows the release to be substantially modified by incorporation of excipients designed to dissolve in aqueous environments. For example, with 40 % NaCl incorporation, release of both peptides was enhanced more than 10-fold. These solids swell and dissolve causing the development of small cracks and pores within the elastomer. This interconnected pathway allows for the release of macromolecules that would otherwise remain trapped in the elastomer network. The rate of release can be controlled by the choice of excipient type and loading. Although in this case, silicone elastomers were used to make the rod inserts, there is no reason why other suitable polymeric systems could not be considered.
